# Uses of the Microscope in the Diagnosis of Cancer

**Published:** 1855-04

**Authors:** 


### *Use of the Microscope in the Diagnosis of Cancer.*

In a recent number of the Gazette des Hopitaux, we find the following sensible view taken of the use of the microscope in cancerous diagnosis :

What are the microscopic characters which have been assigned to cancer? Without entering into the details of microscopic anatomy, we shall content ourselves with indicating the principal results that have been received. A cancerous cell or globule is found, enclosing cellular contents, and one or more nuclei, in which nucleoli is found; but the form of this cancerous globule is far from being constant, being rounded, oval, spherical, often irregular, and presenting prolongations, which have been designated under the name of horus, and which themselves enclose numerous nuclei of cancerous globules. Sometimes even the cancerous cell is wanting, and there are only nuclei enclosing nuclei. Such is the nucleolated cancer. Such are the characters which have been assigned to tumors, designated under the names of scirrhus and encephaloid. But are these the only cancers? No; for there are other tumors, which, like the two last, have the melancholy privilege of recurring, either on the spot, or in the entire economy. Such are the tumors which have been designated under the names of fibro-plastics, of cancroids, and of epithelial tumors. These are not rrue cance s, for elements of another nature have been found in them. In the fibro-plastic tumor are found fusiform globules, lengthened at their extremities, and terminating in true fibres. In epithelial tumors are found cells of which the structure is similar to that of the epidermis or of the epithelium of mucous membrane. Besides these, there are the enchondroma and the melanotic tumors, which also often re-appear with great rapidity, and in which are found elements differing from those just mentioned. Here, then, we have a certain number of tumors, which have all one of the most important clinical characters of cancer—that is to say, which recur, and in which, nevertheless, we do not find the true elements of cancer, that is to say, the cancer cells.

The principal objections, therefore, made to the microscope are, that in the cancer are found cells extremely variable in form. and not always presenting a character of identity; that homœomorphic characters are found in other tumors; and that these tumors sometimes re-appear with as much rapidity and as fatally as true cancer. To these objections the microscopists reply by observing, that no matter what may be the anatomical element of tumors, they have, when they re-appear, their primitive characters; and although some exceptions seem to contradict this theory, we are of opinion that the microscope has been eminently useful in enabling us to make a more accurate anatomical description of such tumors than would have been otherwise possible. In fact. it has indicated their place in nosological classification But, it may be asked, if clinical observation justifies the classification of tumors, as made by the microscope? Yes, in a great number of cases In fact, every surgeon has had opportunities of verifying that the march of cancroids is very different from that of scirrhus and encephaloid cancers True, it matters not which of these tumors it is, the patient always ends by succumbing; but it is to be remarked, that the march of the disease is far from being the same. Of course there are points of identity, since these diseases were for a long time confounded. The same remark may be made of fibro-plastic tumors, which re-appear on the same spot, or in some other part of the economy, but which often present symptoms of a peculiar character.

Another much more serious objection which has boen made to the micrographic examination of epithelial and fibro-plastic tumors, is, that they are composed of elements which exist as well in the economy. Thus, in the adjurated chancre, and in the tissues indurated by inflammation, are found fibro-plastic elements, and yet these latter affections have a march which is entirely their own. They do not recur In warts and syphilitic vegetations, epithelial cells are met with. True, warts recur, as do all vegetations, but this is the one point of contact they have with cancroids; for what can be more dissimilar than those benignant alterationss and an affection which almost always kills? And it is impossible for the microscope to indicate which of these fibro plastic and epithelial affections are susceptible of affecting the economy.

We think, therefore, that instead of merely describing cancerous tumors characterized by a cell of a particular form, there is room for admitting many species of cancer—the fibro-plastic cancer, the epithelial cancer, the encephaloid cancer, the scirrhus cancer, and the melanotic cancer. Each of these species of the genus would have a generic character, that is to say, its re-appearance, and would have, for specific character, the anatomical element established by the microscope. Possibly, the microscope may discover a generic character in the different tumors first enumerated, but it is certainly not the cancerous cell which should be taken as the point of departure; since it does not exist in a certain number of tumors, which are very properly designated under the name of cancer.

Other objections which have been made to microscopic observations, appear to us of no great force. Thus, it has happened, that where one micrograph has established the existence of the cancerous cell, another has not found it. But it may be remarked, that one or more errors on the part of an observer, should not be imputed to the method itself; there are so many causes of error in an instrument requiring such delicate management, that a slight turn of the screw is sufficient to change the whole aspect of the tissues. Besides in doubtful cases, how often do we see two practitioners of equal skill differ in their diagnosis? In such cases, one at least must be wrong, and yet the science of the other may justly remain indisputable.

In the actual state of things, we cannot except all that the microscope asserts; but we may say, that it has already rendered great services, and we do not doubt, that it will continue to do so. It is opened to the reproach of having advanced too far, and of having too readily disregarded clinical observation, although it was there it found the facts which it has used to establish its doctrines. We must add, however, that more recently the attentive examination of facts has enabled it to adopt conclusions more in harmony with the actual development of disease.---*American Med. Monthly.*

